# Impact of PCSK9 inhibitors on lipoprotein(a) levels: a multi-centre study

**DOI:** 10.1093/eurjpc/zwaf734

**Published:** 2025-11-18

**Authors:** Harpreet S. Bhatia, Raphael Cuomo, Mattheus Ramsis, Ehtisham Mahmud, Pam Taub, Michael J. Wilkinson

**Affiliations:** 1 Division of Cardiovascular Medicine, University of California San Diego, 9500 Gilman Drive, MC 7411, La Jolla, CA 92093, USA; 2 School of Medicine, University of California San Diego, La Jolla, CA, USA; 3 Division of Cardiovascular Medicine, University of California San Diego, La Jolla, CA, USA

**Keywords:** Lipoprotein(a), Lipids, PCSK9i

## Abstract

**Aims:**

With no currently available targeted therapies for lipoprotein(a) [Lp(a)] lowering, proprotein convertase subtilisin/kexin type 9 inhibitors (PCSK9i) may be an option for management of increased cardiovascular risk in individuals with elevated Lp(a). However, Lp(a) lowering with PCSK9i is variable. We aimed to evaluate the real-world change in Lp(a) and predictors of response.

**Methods and Results:**

Using data from the University of California Health Data Warehouse, we conducted a multi-centre retrospective study among adults prescribed PCSK9i therapy with available Lp(a) measurement before and after prescription. We evaluated change in Lp(a) compared with baseline and evaluated potential predictors of Lp(a) reduction using multivariable linear regression, including among patients with multiple serial Lp(a) measurements. Among 453 included individuals, PCSK9i use was associated with a median 17.3 [IQR 35.3, 0.0]% and 11.3 [31.7, 0.0] mg/dL reduction in Lp(a) overall. Among those with Lp(a) > 50 mg/dL, a 17.7 [33.6, 0.0]% and 19.2 [42.0, 0.0] mg/dL reduction was observed. Higher baseline Lp(a) level (β −0.31, *P* < 0.001) was a significant predictor of greater Lp(a) reduction, while female sex was associated with less reduction (β 9.28, *P* = 0.02). Results were consistent across Lp(a) assay types and by PCSK9i type and sustained in those with serial Lp(a) measurements (*n* = 274). In contrast, in a control group of 6750 individuals, a median change of 0.00 [−2.00, 3.00] mg/dL in Lp(a) was noted in serial measurements.

**Conclusion:**

PCSK9i are associated with modest Lp(a) lowering of approximately 17% in real-world clinical practice, with a consistent percent reduction by baseline Lp(a) level, PCSK9i type, and Lp(a) assay type. Predictors of Lp(a) reduction include baseline Lp(a) level and sex without significant variation by age, race/ethnicity, or other evaluated comorbidities.

**Lay summary:**

In this study, we evaluated the real-world change in lipoprotein(a) [Lp(a)] after proprotein convertase subtilisin/kexin type 9 inhibitors (PCSK9i) prescription.

- PCSK9i were associated with median 17.3% reduction in Lp(a); in the subset with elevated Lp(a), a similar 17.7% reduction was noted
- Those with higher baseline Lp(a) levels had greater reductions, while women experienced less reduction

## Graphical Abstract

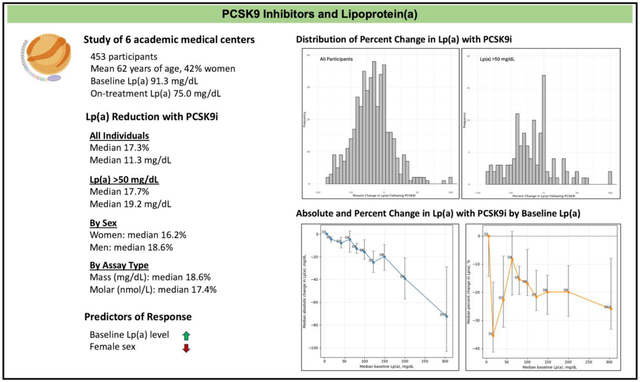

PCSK9 Inhibitors and Lipoprotein(a). In a study of 453 participants from 6 academic medical centres prescribed proprotein convertase subtilisin/kexin type 9 inhibitor (PCSK9) inhibitors with lipoprotein(a) [Lp(a)] measurements before and after, the median reduction in Lp(a) was 17.3% (absolute 11.3 mg/dL) with similar results among those with Lp(a) > 50 mg/dL and by assay type, with variation by sex. Baseline Lp(a) was a predictor of greater response, while female sex was a predictor of less response. On the top right, histograms of pre-post percent change in Lp(a) following PCSK9 inhibitory therapy, as measured in milligrams per decilitre, with 5 unit percentage bin widths, are shown among all participants and among those with Lp(a) > 50 mg/dL. On the bottom right, median baseline Lp(a) was plotted against median absolute and median per cent change (95% CI) in Lp(a) following PCSK9 inhibitor therapy for all deciles of baseline Lp(a).

## Introduction

Elevated lipoprotein(a) [Lp(a)] is a genetic risk factor for atherosclerotic cardiovascular disease (ASCVD) and aortic stenosis.^1^ Despite being a common risk factor, present in approximately 20% of the global population, testing rates for Lp(a) are very low.^2^ One barrier to more widespread Lp(a) testing is uncertainty among physicians regarding the clinical management of patients with elevated Lp(a), and the lack of currently available targeted therapies for Lp(a) lowering.

However, proprotein convertase subtilisin/kexin type 9 inhibitors (PCSK9i) have been shown to modestly reduce Lp(a) levels in addition to potent lowering of low-density lipoprotein–cholesterol (LDL-C)^3,4^ and may be an option for management of patients with elevated Lp(a) until targeted therapies are available. Evolocumab was associated with a median reduction in Lp(a) levels of 11 nmol/L or 27% over 48 weeks in a secondary analysis of the FOURIER trial,^3^ and alirocumab was associated with a median reduction of 5 mg/dL (approximately 12.5 nmol/L) or 23% over 4 months in a secondary analysis of the ODYSSEY OUTCOMES trial.^4^ In both cases, the reduction in Lp(a) levels appeared to translate into reduction in cardiovascular risk.^3,4^

However, neither trial was designed to specifically address this question and the patient populations were not enriched with individuals with elevated Lp(a) levels. Additionally, there is significant heterogeneity in the response of Lp(a) levels to PCSK9i in clinical practice.

We aimed to perform a real-world study to evaluate the response to Lp(a) levels with PCSK9i and to evaluate for potential clinical predictors of Lp(a) response to guide targeted clinical management.

## Methods

### Study design

We conducted a retrospective cohort study using data from the University of California Health Data Warehouse (UCHDW) which contains harmonized electronic health record data from six academic medical centres associated with the University of California, in accordance with STROBE guidelines. For this study, all individuals ≥18 years of age prescribed a PCSK9i (evolocumab, alirocumab, or inclisiran) who had at least one Lp(a) measurement before and after PCSK9i prescription were included from 2012 to March 2025. For quality control, we evaluated the change in LDL-C with PCSK9i, comparing LDL-C within 1 year prior to PCSK9i therapy and at least 30 days following initiation of therapy. Participants without any reduction in LDL-C and those with only a single PCSK9i prescription were excluded for possible non-adherence or early discontinuation of therapy. We included a separate control group of individuals with two Lp(a) measurements at least 30 days apart who were not prescribed PCSK9i. Lp(a) measurements in both mass (mg/dL) and molar (nmol/L) units were available; molar units were converted to mass units using a conversion factor of 0.4167.^5^ Given that both measurement types are used clinically, results are presented in both milligrams per decilitre and nanomoles per litre. LDL-C values were calculated using the Friedwald equation. All patient data were deidentified, and the study was approved by the institutional review board under a data use agreement for retrospective analysis of limited datasets.

### Statistical analysis

Baseline characteristics were compared by Lp(a) level among those prescribed and not prescribed PCSK9i therapy. Continuous variables were compared using Student’s *t*-tests or Mann–Whitney *U* tests, and categorical variables were compared using χ^2^ testing.

We evaluated the change in Lp(a) after PCSK9i therapy using paired *t*-tests. We then evaluated potential predictors of change in Lp(a) level with PCSK9i therapy including age, sex, race/ethnicity, body mass index (BMI), diabetes, hypertension, smoking history, total cholesterol, high-density lipoprotein–cholesterol (HDL-C), triglycerides, and race-specific estimated glomerular filtration rate (eGFR) in multivariable linear regression models. Missing data were handled using complete case analysis, and model residuals were examined for normality and homoscedasticity. Results from these models were reported as estimated beta coefficients with 95% confidence intervals and associated *P*-values. These analyses were performed in all participants, regardless of the Lp(a) level, as well as the subgroup of those with elevated Lp(a) > 50 mg/dL. Sensitivity analyses were performed to determine the influence of assay type, high-sensitivity C-reactive protein (hsCRP), statin intensity, hypothyroidism, and sex-specific differences.

We then evaluated for sustained changed in Lp(a) among individuals with at least three Lp(a) measurements by comparing the second Lp(a) measurement to the mean of all subsequent measures (third and beyond) using paired *t*-tests.

All analyses were performed in R (version 4.2.2), and figures were generated using the ggplot2 package. A *P*-value <0.05 was considered statistically significant.

## Results

A total of 453 individuals were included in the PCSK9i study cohort, and 6750 controls not prescribed PCSK9i were included. Baseline characteristics of participants by the Lp(a) level and PCSK9i prescription are shown in Table 1. Among those prescribed PCSK9i, those with Lp(a) > 50 mg/dL had less LDL-C reduction, greater absolute reduction in Lp(a) and similar percent reduction in Lp(a).

The median baseline Lp(a) was 91.26 [IQR 41.67, 149.00] mg/dL (219.02, IQR 100.01, 357.60 nmol/L) and median follow-up was 75.00 [29.00, 119.00] mg/dL or 180 [69.60, 285.60] nmol/L (*P* < 0.001). The median absolute reduction was 11.25 [31.67, 0.00] mg/dL (27.00; 76.01, 0.00 nmol/L), corresponding to median per cent reduction in Lp(a) of 17.4 [35.3, 0.0]. Exclusion of participants prescribed inclisiran produced similar results. The median time between PCSK9i initiation and post-treatment measurement was 116 [70, 247] days. The median interval between pre- and post-treatment Lp(a) values was 232 [131, 655] days. The absolute median Lp(a) reduction for women was 12.29 (31.75, 1.92) mg/dL (29.50; 76.20, 4.61 nmol/L) vs. 11.00 [31.46, 0.00] mg/dL (26.40; 75.51, 0.00 nmol/L) for men (*P* = 0.578). The median per cent reduction for women was 16.2 [33.3, 2.2] vs. 18.6 [36.4, 0.0] for men (*P* = 0.077). The median per cent reduction among those prescribed evolocumab was 17.7 [34.7, 0.0]% and among those prescribed alirocumab was 16.5 [33.0, 0.9] (*P* = 0.349). The median per cent reduction in LDL-C for the full sample was 51.8%, with an interquartile range of 32.11–68.39%, consistent with expected pharmacodynamic response to PCSK9i. Among those with Lp(a) > 50 mg/dL (120 nmol/L, *n* = 330), the median absolute reduction was 19.17 [42.04, 0.00] mg/dL (46.01; 100.90, 0.00 nmol/L) and percent reduction was 17.7 [33.6, 0.0]. At baseline, 327 (72.2%) participants had Lp(a) > 50 mg/dL compared with 301 (66.4%) after PCSK9i prescription (*P* = 0.061). A histogram of per cent change in Lp(a) is shown in all participants and among only those with Lp(a) > 50 mg/dL in the *Graphical Abstract*, illustrating significant heterogeneity in response. By comparison, control participants experienced a median absolute change in Lp(a) of 0.00 [−2.00, 3.00] mg/dL and median per cent change of −1.23 [−14.97, 13.27]%.

For the analysis of predictors of Lp(a) change with PCSK9i, 380 participants in the PCSK9i cohort had complete covariate date for inclusion. In the multivariable model, baseline Lp(a) was significantly associated with change in Lp(a) following PCSK9i initiation (β −0.31, SE = 0.02, *P* < 0.001), indicating that individuals with higher baseline Lp(a) values experienced larger absolute reductions. Additionally, female sex was associated with less Lp(a) reduction (β 9.28, SE = 4.84, *P* = 0.02) compared with male sex. When restricted to those with Lp(a) > 50 mg/dL (*n* = 263), similar results were seen regarding baseline Lp(a) (β −0.36, SE = 0.03, *P* < 0.001) with a similar trend for female sex (β 11.70, SE = 6.80, *P* = 0.06, Table 2). In the overall cohort including individuals prescribed PCSK9i and control participants without PCSK9i prescription, PCSK9i therapy was independently associated with a reduction in Lp(a) levels overall (β −16.26, SE = 1.41, *P* < 0.001) and among those with Lp(a) > 50 mg/dL only (β −22.10, SE = 2.60, *P* < 0.001, Table 2). Median absolute change in Lp(a) by the baseline Lp(a) level is shown in the *Graphical Abstract*, demonstrating a linear relationship between baseline Lp(a) and change in Lp(a) with PCSK9i therapy. For Lp(a) levels above the second decile (15.4 mg/dL or 36.96 nmol/L), a relatively flat per cent reduction in Lp(a) was observed by baseline Lp(a) (*Graphical Abstract*). As a sensitivity analysis, additional adjustment for hsCRP was made in individuals with available measurements (*n* = 249); hsCRP was not associated with change in Lp(a) and additional adjustment did not affect the association between baseline Lp(a) and Lp(a) change. Similarly, intensity of statin therapy and hypothyroidism were not associated with change in Lp(a) levels.

As a sensitivity analysis, those with Lp(a) measured by a mass (mg/dL) assay (*n* = 183) and by a molar (nmol/L) assay (*n* = 262) were evaluated separately. Among those with measurement in mass units, the median baseline and post-treatment values were 112.00 [48.00, 186.5] mg/dL and 87.00 [34.00, 150.00] mg/dL, respectively (*P* < 0.001), with a median absolute reduction of 15.00 [37.00, 1.00] mg/dL, corresponding with a median 18.6 [34.9, 3.5]% reduction. Among those with measurement in molar units, the median baseline Lp(a) concentration was 205.50 [IQR 99.25, 310.50] nmol/L, which declined significantly to 174.50 [58.05, 261.25] nmol/L post-treatment (*P* < 0.001), corresponding to a median absolute reduction of 22.00 [71.25, 1.00] nmol/L and median per cent reduction of 17.4 [35.7, 0.6]%. In multivariable adjusted models, baseline Lp(a) remained significantly associated with greater Lp(a) reduction for both assay types (*P* < 0.001). In the mass assay group, greater eGFR was also significantly associated with greater reduction in Lp(a) (β −0.66, *P* = 0.041). In the molar assay group, the presence of diabetes was associated with greater Lp(a) reduction (β −55.10, *P* = 0.008).

**Figure 1:**
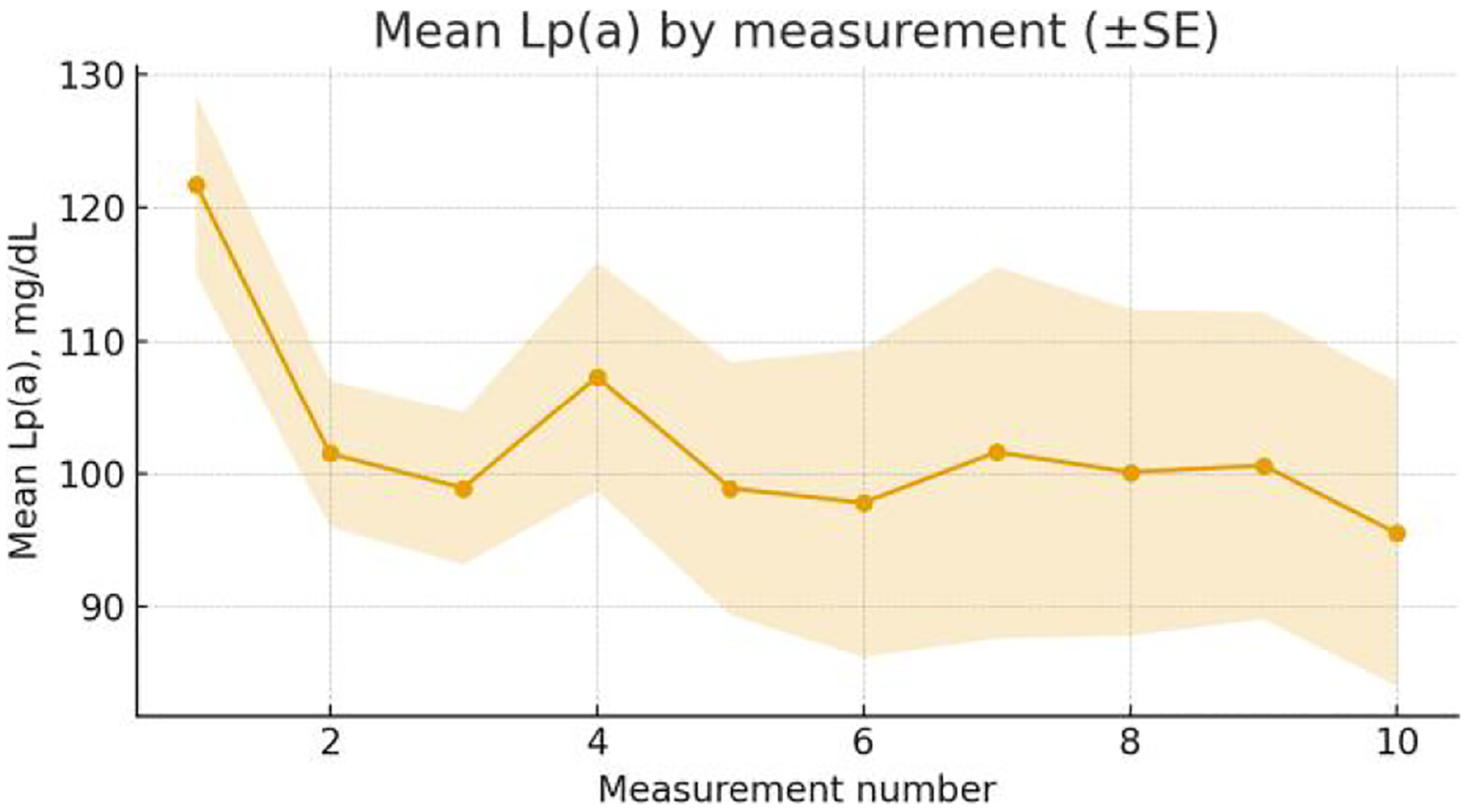
Change in Lp(a) with PCSK9i therapy over repeated measurements. Mean (SE) Lp(a) values (mg/dL) for each sequential measurement from the first through the 10th among 274 participants who had at least three recorded Lp(a) measurements. When comparing the second Lp(a) measurement to the mean of all subsequent (third or later) measurements, the mean absolute difference was −1.57 (37.34) mg/dL (*P* = 0.489).

To examine whether Lp(a) reductions were sustained over time, we performed a longitudinal analysis restricted to the 274 patients who had at least three Lp(a) measurements. For these patients, the mean Lp(a) value declined from 121.7 mg/dL (SD 111.8) or 292.1 (268.3) nmol/L at the first measurement to 101.5 mg/dL (91.2) or 243.6 (218.9) nmol/L at the second (median 97 [52, 200] days) and to 98.9 mg/dL (94.8) or 237.4 (227.5) nmol/L at the third (median 305 [174, 637] days). Means were then calculated for each successive measurement, revealing mild fluctuations but no consistent upward rebound (Figure 1). Across the full sequence, mean Lp(a) values remained below baseline through the 10th measurement, up to 1008 [604, 1225] days (see Supplementary material online, Table S1). When comparing the second Lp(a) measurement to the mean of all subsequent (third or later) measurements, the mean absolute difference was −1.57 (37.34) mg/dL or −3.77 (89.62) nmol/L (*P* = 0.489), suggesting that the drop in Lp(a) from the first to second measure was sustained across further measures.

## Discussion

In a large, multi-centre, real-world study, the use of PCSK9i resulted in modest lowering of Lp(a) of 17.3% among all participants, with a similar 17.7% reduction among those with Lp(a) > 50 mg/dL (>120 nmol/L). This response was highly variable, including among those with elevated Lp(a). The strongest predictor of Lp(a) response overall was the baseline Lp(a) level—those with higher Lp(a) levels experienced greater absolute reductions in Lp(a) and the percentage reduction was relatively consistent across Lp(a) levels. Women also experienced less Lp(a) reduction than men. Overall results were consistent with measurement using mass and molar assays, by PCSK9i type, and with adjustment for hsCRP.

The initial studies evaluating the effect of PCSK9i on Lp(a) levels were secondary analyses of clinical trials. In an analysis of the FOURIER trial, which evaluated evolocumab vs. placebo in 25 096 individuals with prior ASCVD, the median Lp(a) reduction was 27% at 48 weeks. This reduction was highly variable, with an interquartile range of 6–47%. There was a trend towards greater improvement in outcomes with PCSK9i among those with higher Lp(a) levels, and the reduction in Lp(a) was linearly associated with the reduction in risk.^3^ In an analysis of the ODYSSEY OUTCOMES trial, which evaluated alirocumab vs. placebo in 18 924 individuals with recent acute coronary syndrome, there was a 23% reduction in Lp(a) at 4 months, again with a variable response with an interquartile range of 0–47%. A greater reduction in Lp(a) was noted with higher baseline Lp(a) and a higher baseline Lp(a) level predicted a greater reduction in risk with PCSK9i therapy.^4^ Another analysis of ODYSSEY OUTCOMES observed that in individuals with LDL-C near 70 mg/dL, there was incremental benefit to PCSK9i therapy when Lp(a) levels were higher.^6^ However, these trials were not designed to specifically address the effects of PCSK9i on Lp(a) and were not enriched with individuals with elevated Lp(a).

In a meta-analysis of PCSK9i randomized controlled trials with 67 057 individuals, mean reduction in Lp(a) was 27%, and per cent change in LDL-C and apoB were associated with Lp(a) reduction.^7^ A limitation of this analysis is that both LDL-C and apoB overlap with Lp(a) levels. Lp(a) is an apoB containing lipoprotein, and LDL-C is calculated and includes Lp(a)-cholesterol. Thus, the association of LDL-C and apoB with Lp(a) reduction may be due to inclusion of measures of Lp(a) in these markers. Another recent meta-analysis of randomized controlled trials demonstrated a reduction in Lp(a) with PCSK9i of 29% with monoclonal antibodies and 22% with inclisiran. Specific predictors of response were not evaluated, but a greater absolute reduction in Lp(a) was noted with higher baseline Lp(a).^8^ Few prior studies have evaluated Lp(a) lowering with PCSK9i specifically among those with elevated Lp(a) levels. In a small randomized controlled trial of 129 individuals with median Lp(a) of 200 nmol/L (80 mg/dL), mean reduction in Lp(a) was 13.9% (95% CI 8.5–19.3%).^9^ Finally, in a prospective study of 150 participants with LDL-C > 100 mg/dL and median Lp(a) of 27.5 mg/dL, a median 10 [IQR 0, 36]% reduction in Lp(a) was observed with evolocumab. There was greater Lp(a) lowering of 15% among those with Lp(a) ≥ 30 mg/dL (*n* = 72). The larger apolipoprotein(a) isoform size was associated with greater per cent reduction in Lp(a), and the baseline Lp(a) level was not associated when also adjusted for the apolipoprotein(a) size. Additionally, Black race/ethnicity, age, and LDL-C level were also positively associated with Lp(a) reduction.^10^

Our study adds several clinically relevant aspects to the literature. In a large real-world study, Lp(a) lowering with PCSK9i is modest compared with clinical trials, and the response is highly variable. Although underlying mechanisms may differ, heterogeneity in treatment response for LDL-C has also been observed in real-world studies of PCSK9i monoclonal antibodies and small interfering RNA therapies (siRNA).^11–13^ Notably, the per cent reduction in Lp(a) was similar in those with elevated Lp(a), translating to greater absolute reduction in Lp(a), which is in line with the ODYSSEY OUTCOMES analysis. Additionally, this modest reduction is much less than the estimated Lp(a) lowering needed for clinically meaningful improvement in outcomes: Prior studies have estimated an absolute reduction in Lp(a) of 65.7–101.5 mg/dL is needed to achieve the same risk reduction as a 38.7 mg/dL lowering of LDL-C.^14^ By comparison, novel Lp(a)-targeted therapies are estimated to achieve >80–90% reductions in Lp(a) levels.^15^ However, Lp(a) is recognized as a continuous risk factor for cardiovascular disease and in the analysis of ODYSSEY OUTCOMES, a 1 mg/dL reduction in Lp(a) was associated with a 0.6% reduction in risk.^4^ This suggests that the observed 19 mg/dL absolute reduction in Lp(a) among those with Lp(a) > 50 mg/dL may still be clinically meaningful. Additionally, there appears to be greater overall benefit to PCSK9i in those with higher Lp(a) levels.^6^ Given the lack of available targeted therapies for Lp(a) lowering, our study adds to the existing literature suggesting that PCSK9i are the best currently available medications for addressing Lp(a)-mediated risk.

We additionally evaluated predictors of Lp(a) response, and prior literature addressing this area has been limited. The best predictor of Lp(a) response overall was the baseline Lp(a) level. In a prior smaller study evaluating Lp(a) response with PCSK9i, baseline Lp(a) did not predict response,^10^ but this was after adjusting for the apolipoprotein(a) isoform size which is associated with Lp(a) levels. Additionally, female participants had less Lp(a) lowering than male participants. The underlying reasons for this are unclear. However, prior studies have also demonstrated less LDL-C lowering with PCSK9i in women vs. men.^16^ A potential explanation is that women may have higher circulating levels of PCSK9 than men^17^ and thus may respond less to PCSK9 inhibition. It is well established that Lp(a) levels are higher in women than in men.^18^ Additionally, Lp(a) levels rise in women with age, particularly after menopause.^19^ In the present study, regression models were adjusted for baseline Lp(a) levels, but it is possible that the lower response to PCSK9i therapy in women is related to hormonal influences. Further study is needed to better understand this association.

The eGFR was also a significant predictor of response in the mass assay group, and diabetes was a significant predictor in the molar assay group. Further studies are needed to understand the underlying physiology, but it is established that impaired renal function leads to decreased clearance of Lp(a).^20^ Notably, LDL-C reduction with evolocumab is consistent across stages of chronic kidney disease studied.^21^ The interaction between Lp(a) and diabetes has been the subject of much debate; larger apolipoprotein(a) isoforms may be associated with diabetes.^22^ As noted above, prior studies have observed greater Lp(a) reduction with PCSK9i with a larger apolipoprotein(a) isoform size.^10^ which may explain the association observed in the current study. It is unclear why these associations varied by mass vs. molar assay measurement. These findings should be interpreted with caution and considered hypothesis generating given the small sample size in subgroups. Our study also demonstrated consistency in Lp(a) lowering across Lp(a) assay types and with both evolocumab and alirocumab without a significant impact of age, race/ethnicity, or other medical comorbidities. Lp(a) lowering was sustained over time in individuals with serial Lp(a) measurements which has not been previously described, to our knowledge, with longer follow-up than in the prior secondary analysis of clinical trials. In particular, serial measurements were available in the majority of participants, demonstrating sustained Lp(a) reduction up to a median of 305 days and up to 1008 days in a subset or participants. Finally, a consistent, independent association between PCSK9i prescription and Lp(a) reduction was demonstrated when including control participants.

Our study has notable limitations. Despite being a multi-centre study, the sample size was relatively small which may have limited the ability to detect significant differences among subgroups, in particular. However, to our knowledge, this study was one of the largest real-world descriptions of PCSK9i and Lp(a). As an observational study, we were not able to confirm adherence to PCSK9i therapy; however, we attempted to address this by excluding those with only a single prescription and those without LDL-C lowering response to therapy. Additionally, the observed 51.8% reduction in LDL-C is in line with the expected reduction with PCSK9i therapy. Few participants were prescribed inclisiran, which limited our ability to evaluate for the specific response to inclisiran. We are also unable to determine the reason for Lp(a) testing as measurements were conducted in the course of usual clinical care; however, 79% of participants in this study had a history of ASCVD and likely represent a higher-risk population. While we do not suspect that the results for Lp(a) lowering would be different in other populations with similar Lp(a) levels, further study may be needed to generalize these results. Additionally, there is no standardized factor for converting between Lp(a) levels in mass and molar units. Though we observed consistent results by the Lp(a) assay type, heterogeneity in Lp(a) assays and lack of a standardized Lp(a) measurement remain limitations. Finally, evaluation of predictors of Lp(a) response was limited to variables available in the electronic health record. There may be other predictors of importance such as the apolipoprotein(a) isoform size which has previously been demonstrated. However, this is not readily available clinically, and Lp(a) levels are closely correlated with the isoform size.

In summary, PCSK9i are associated with modest Lp(a) lowering in real-world clinical practice, with a consistent percent reduction by the baseline Lp(a) level, PCSK9i type, and assay type. Predictors of absolute Lp(a) reduction in response to PCSK9i therapy include baseline Lp(a) and sex without significant variation by age, race/ethnicity, or other evaluated comorbidities.

## Supplementary Material

Supplementary Material

Supplementary material is available at European Journal of Preventive Cardiology.

## Figures and Tables

**Table 1:** Baseline characteristics of study participants by the lp(a) level

	PCSK9i cohort		*P*	Control cohort		*P*
	Lp(a) ≤ 50 mg/dL (*n* = 128)	Lp(a) > 50 mg/dL (*n* = 330)		Lp(a) ≤ 50 mg/dL (*n* = 4429)	Lp(a) > 50 mg/dL (*n* = 2322)	
Age, years	63.52 (11.47)	61.97 (11.56)	0.20	59.01 (15.73)	56.13 (16.32)	<0.001
Female sex	43 (33.59)	148 (44.85)	0.03	2019 (44.62)	1160 (48.54)	<0.001
Race/ethnicity						
Asian	14 (10.94)	36 (10.91)	0.99	390 (8.62)	212 (8.87)	0.72
Black	1 (0.78)	11 (3.33)	0.19	73 (1.61)	124 (5.19)	<0.001
Hispanic/Latino	4 (3.12)	14 (4.24)	0.79	347 (7.67)	162 (6.78)	0.18
Native American	0 (0.00)	1 (0.30)	1.00	14 (0.31)	1 (0.04)	0.03
White	100 (78.12)	235 (71.21)	0.13	3315 (73.26)	1634 (68.37)	<0.001
Multiple	3 (2.34)	8 (2.42)	1.00	159 (3.51)	95 (3.97)	0.33
BMI, kg/m^2^	26.89 (5.10)	26.32 (4.56)	0.28	27.60 (56.13)	26.56 (5.58)	0.24
History of ASCVD	110 (85.94)	253 (76.67)	0.03	1908 (42.17)	1106 (46.28)	<0.001
Diabetes	22 (17.19)	30 (9.09)	0.01	389 (8.60)	225 (9.41)	0.26
Hypertension	87 (67.97)	217 (65.76)	0.65	2193 (48.46)	1214 (50.79)	0.07
Smoking history	3 (2.34)	9 (2.73)	1.00	129 (2.85)	70 (2.93)	0.85
Statin use	113 (88.28)	314 (95.15)	0.01	3055 (67.51)	1787 (74.77)	<0.01
eGFR, mL/min/1.73 m^2^	75.04 (17.08)	75.88 (15.48)	0.64	75.71 (18.25)	74.38 (18.63)	0.01
Total cholesterol, mg/dL	155.50 [123.00, 193.75]	145.00 [114.00, 176.00]	0.05	176.00 [144.00, 213.00]	176.00 [146.00, 211.00]	0.84
Triglycerides, mg/dL	93.00 [68.00, 133.75]	79.00 [60.00, 114.00]	<0.001	90.00 [66.00, 131.00]	85.00 [63.00, 119.00]	<0.001
HDL-C, mg/dL	51.00 [43.75, 63.25]	56.50 [47.00, 68.75]	0.01	56.00 [45.00, 70.00]	57.00 [46.00, 70.00]	0.18
Baseline LDL-C, mg/dL	108.10 [79.95, 144.65]	85.80 [70.00, 117.55]	<0.001	105.90 [76.80, 136.80]	101.00 [73.40, 133.60]	<0.001
On-treatment LDL-C, mg/dL	54.10 [33.35, 78.75]	43.50 [25.20, 66.55]	<0.001	—		
LDL-C change, mg/dL	−52.60 [28.40, 74.40]	−44.40 [26.55, 62.60]	0.05	—		
Baseline Lp(a), mg/dL	16.00 [6.81, 27.71]	119.59 [84.15, 178.76]	<0.001	10.83 [6.00, 23.34]	92.72 [67.51, 138.00]	<0.001
On-treatment Lp(a), mg/dL	9.79 [6.00, 23.75]	97.92 [66.13, 144.39]	<0.001	—		
Lp(a) change, mg/dL	−2.00 [−8.00, 0.00]	−19.17 [−42.04, 0.00]	<0.001	—		
Lp(a) change, %	−16.66 [−38.11,0.00]	−17.68 [−33.65, 0.00]	0.82	—		
PCSK9i						
Evolocumab	108 (84.38)	287 (86.97)	0.47	—		
Alirocumab	50 (39.06)	115 (34.85)	0.40	—		
Inclisiran	1 (0.78)	8 (2.42)	0.46	—		

Data presented as mean (SD), median [IQR] or *n* (%).

**Table 2:** Predictors of change in lp(a) with PCSK9i

	PCSK9i Cohort						All participants					
	All participants (*n* = 380)			Lp(a) > 50 mg/dL (*n* = 263)			All participants (*n* = 5520)			Lp(a) > 50 mg/dL (*n* = 2044)		
Variable	β	Std. Error	*P*	β	Std. Error	*P*	β	Std. Error	*P*	β	Std. Error	*P*
Age, years	−0.29	0.23	0.20	−0.48	0.32	0.14	−0.05	0.03	0.11	−0.11	0.07	0.12
Female sex	9.28	4.84	0.02	11.70	6.80	0.06	2.31	0.77	<0.001	6.13	1.86	<0.001
Race/ethnicity												
Asian	−2.82	7.18	0.70	−2.49	10.2	0.81	0.31	1.25	0.80	−0.97	3.01	0.75
Black	8.84	13.3	0.66	6.02	16.8	0.72	6.16	2.04	<0.001	4.19	3.75	0.26
Hispanic/Latino	0.71	17.3	0.97	0.49	22.10	0.98	−5.97	2.13	0.01	−13.57	5.19	0.01
Native American	20.80	41.1	0.61	12.20	48.6	0.82	0.55	8.50	0.95	31.19	37.54	0.41
Other/Unknown	6.26	7.17	0.74	10.30	9.80	0.30	−2.18	1.09	0.05	−3.08	2.61	0.24
White	REF	—	—	REF	—	—	REF	—	—	REF	—	—
Diabetes	−3.05	6.76	0.65	−2.97	11.00	0.79	−0.80	1.18	0.50	−3.53	2.80	0.21
Hypertension	−0.07	4.84	0.99	2.81	6.76	0.68	1.77	0.76	0.02	4.08	1.90	0.03
Smoking history	5.02	12.6	0.69	3.85	17.70	0.83	−2.31	1.90	0.22	−8.34	4.53	0.07
Body mass index, kg/m^2^	0.77	0.52	0.14	1.10	0.75	0.14	0.00	0.01	0.56	0.15	0.17	0.39
Estimated GFR, mL/min/1.73 m^2^	−0.13	0.14	0.35	−0.32	0.21	0.13	−0.03	0.02	0.11	−0.09	0.05	0.08
Total cholesterol, mg/dL	0.06	0.05	0.24	0.08	0.08	0.28	0.02	0.01	0.02	0.05	0.02	0.03
HDL-C, mg/dL	−0.01	0.16	0.97	0.02	0.23	0.92	−0.08	0.02	<0.001	−0.19	0.06	<0.001
Triglycerides, mg/dL	−0.02	0.03	0.51	−0.02	0.04	0.58	−0.01	0.00	0.03	−0.02	0.01	0.07
Baseline Lp(a), mg/dL	−0.31	0.02	<0.001	−0.36	0.03	<0.001	−0.11	0.01	<0.001	−0.16	0.01	<0.001
PCSK9i therapy	—						−16.26	1.41	<0.001	−22.10	2.60	<0.001

Results from a multivariable linear regression model assessing predictors of absolute change in Lp(a) levels following initiation of PCSK9i inhibitor therapy in 380 participants prescribed PCSK9i overall, including 263 participants with Lp(a) > 50 mg/dL with complete baseline covariate data. Predictors of change in Lp(a) levels in all participants, including controls not prescribed PCSK9i therapy, are shown to the right, demonstrating the independent association between PCSK9i therapy and Lp(a) reduction.

## Data Availability

The data that support the findings of this study are derived from the University of California Health Data Warehouse (UCHDW), which consists of deidentified patient information. Due to the sensitive nature of the clinical data and the agreements under which they were obtained, the data cannot be made publicly available.
