# Supplementary material for: Impact of PCSK9 inhibitors on lipoprotein(a) levels: a multi-centre study
Source: Eur J Prev Cardiol. Author manuscript; Available in PMC 2026 Jul 13. (PMC13361851; doi:10.1093/eurjpc/zwaf734)
Supplement: Supplementary Material [file NIHMS2191422-supplement-Supplementary_Material.docx]

**Supplemental Table 1.** Change in Lp(a) with PCSK9i therapy over repeated measurements

| **Measurement number** | **n** | **Median (IQR) days from PCSK9i prescription** | **Mean Lp(a) (SD)** |
| --- | --- | --- | --- |
| 1 | 274 | -62 (-172, -22) | 121.7 (111.8) |
| 2 | 274 | 97 (52, 200) | 101.5 (91.2) |
| 3 | 274 | 305 (174, 637) | 98.9 (94.8) |
| 4 | 134 | 534 (323, 896) | 107.3 (99.6) |
| 5 | 75 | 681 (436, 978) | 98.9 (82.4) |
| 6 | 55 | 857 (502, 1331) | 97.8 (85.9) |
| 7 | 37 | 936 (617, 1220) | 101.6 (85.0) |
| 8 | 25 | 943 (813, 1569) | 100.1 (61.3) |
| 9 | 19 | 1027 (756, 1550) | 100.6 (50.3) |
| 10 | 12 | 1008 (604, 1225) | 95.5 (39.7) |

Mean (SD) Lp(a) values (mg/dL) for each sequential measurement from the first through the tenth among 274 participants who had at least three recorded Lp(a) measurements, including number of patients contributing to each measurement. When comparing the second Lp(a) measurement to the mean of all subsequent (third or later) measurements, the mean absolute difference was -1.57 (37.34) mg/dL (p=0.489).
